# Cell wall microstructure, pore size distribution and absolute density of hemp shiv

**DOI:** 10.1098/rsos.171945

**Published:** 2018-04-04

**Authors:** Y. Jiang, M. Lawrence, M. P. Ansell, A. Hussain

**Affiliations:** BRE Centre for Innovative Construction Materials, Department of Architecture and Civil Engineering, University of Bath, Bath BA2 7AY, UK

**Keywords:** hemp shiv, microstructure, porosity, absolute density, mercury intrusion porosimetry, helium pycnometry

## Abstract

This paper, for the first time, fully characterizes the intrinsic physical parameters of hemp shiv including cell wall microstructure, pore size distribution and absolute density. Scanning electron microscopy revealed microstructural features similar to hardwoods. Confocal microscopy revealed three major layers in the cell wall: middle lamella, primary cell wall and secondary cell wall. Computed tomography improved the visualization of pore shape and pore connectivity in three dimensions. Mercury intrusion porosimetry (MIP) showed that the average accessible porosity was 76.67 ± 2.03% and pore size classes could be distinguished into micropores (3–10 nm) and macropores (0.1–1 µm and 20–80 µm). The absolute density was evaluated by helium pycnometry, MIP and Archimedes' methods. The results show that these methods can lead to misinterpretation of absolute density. The MIP method showed a realistic absolute density (1.45 g cm^−3^) consistent with the density of the known constituents, including lignin, cellulose and hemi-cellulose. However, helium pycnometry and Archimedes’ methods gave falsely low values owing to 10% of the volume being inaccessible pores, which require sample pretreatment in order to be filled by liquid or gas. This indicates that the determination of the cell wall density is strongly dependent on sample geometry and preparation.

## 1. Introduction

Bio-based insulation materials, such as hemp, flax and wheat straw, offer a number of benefits in comparison with more established mineral and oil-based alternatives, such as mineral wool and PUR (polyurethane rigid foam)-based products [1,2]. Apart from timber, hemp-lime is perhaps the most researched bio-based building material because it is a cheap and low density material (bulk density: 0.08–0.16 g cm^−3^) with associated low thermal conductivity (0.06–0.14 W k^−1^ m^−1^) depending on the density and moisture level. Hemp-lime (often referred to a ‘hempcrete’) is a composite material made up of the woody core of the hemp stalk (shiv) used as an aggregate in a lime-based binder. The binders are typically based on hydraulic lime but specially formulated to avoid the inhibition of hydration caused by the sugars present in the hemp stalk.

Previous research on the physical properties of hemp-lime has demonstrated that the material presents a good balance between low mass and heat storage capacity compared with classical insulation materials. Much of the existing characterization data for natural building materials relates to structural performance such as cell wall microstructure and porosity [3–9]. Thus, concise knowledge of the cell wall microstructure, porosity and the absolute density is of high importance for the characterization of bio-aggregated materials. However, the characterization of the properties of bio-based building materials is at an early stage.

**Figure 1.**
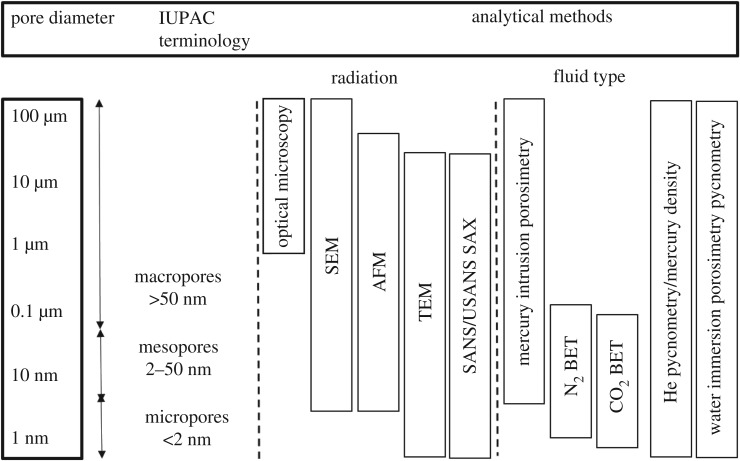
Methods used to determine porosity and pore size distribution (PSD) (reproduced from [19]).

To the best of our knowledge, there is no detailed study on the cell wall ultrastructure, pore size distribution and absolute density of hemp shiv (woody core). The objective of this paper is to establish the most appropriate techniques that should be used to identify the characteristic qualities of hemp shiv and to present representative data for hemp shiv typically used in construction. This is because hemp shiv is, by far, the most commonly used bioaggregate in the construction industry and the information provided in this paper will be of great use to researchers and practitioners alike. Recently, several studies investigated the microstructure of hemp shiv. Magniont *et al*. [1] reported that hemp shiv was embedded in resin and polished and a cross section of hemp shiv was studied by scanning electron microscopy (SEM). They showed that hemp shiv is mainly composed of conducting vessels. The diameter of the vessels usually ranged from 10 to 20 µm. In addition, larger vessels were also observed in a longitudinal section [1]. Lawrence *et al.* [10] found the microstructure of hemp shiv exhibited 50 µm pores connected to 10 µm pores via 1 µm connecting pores. Dubois *et al*. [11] showed a section through one hemp shiv obtained by X-ray microtomography. The high total porosity of the vegetal particle can be clearly seen with the typical tubular structure of the mesoscopic pores. The porosity of hemp shiv is quite a simple parameter to define but is not so easy to quantify. The reason is that the void/space in hemp shiv can span from a few nanometres to millimetres or larger. There is currently no one measurement method that can adequately cover this enormous range in scale. In addition, the porosity can be modified or changed by a variety of processes during the porosity measurement such as deformation, hydrothermal alteration and the production of secondary or fracture porosity. Finally, the pore shape and connection structure (open and closed) have a significant effect on the porosity results depending on the testing approach [12–18]. Anovitz *et al*. [19] summarized 10 methods for measuring the porosity and pore size distribution (PSD) used on core or crushed rock materials (figure 1), revealing the range of pore sizes that each method is capable of measuring. It should be kept in mind that different techniques are based on different principles and have different capabilities for measurement. Depending on the natural properties of bio-aggregates, there is no best approach to determine their porosity. The combination of several techniques and comparison of results of pore structure investigations from different methods may give an insight into the complex porosity of bio-aggregates.

Similarly, the determination of cell wall density of (porous material) hemp shiv is not a simple matter. The essential requirement is that all pore spaces are freely accessible for the displacement gas or liquid. This means that all cell lumens within the hemp shiv, such as vessels and voids in the cell wall, must be reached by the displacement gas or liquid. Gas pycnometry is a very accurate method for measuring the absolute density of cell walls, which is based on the Archimedes' principle of displacement of an inert gas, such as helium, nitrogen or argon. Although the method of gas pycnometry is relatively simple compared with other methods, there are only very few publications dealing with investigations of the cell wall density or porosity of hemp shiv using this procedure, owing to the complicated sample preparation. Hill *et al*. [20] prepared samples of pine using a Soxhlet extraction for 8 h with a solvent system composed of toluene, methanol and acetone (4 : 1 : 1 by volume). Samples were then oven-dried for 12 h at 105°C and cooled in a desiccator before placing into a helium pycnometer. The cell wall density of the untreated pine sample was 1.42 g cm^−3^. Zauer *et al*. [21] studied the cell wall density of wood determined by gas pycnometry. The results clearly showed that misinterpretation of the results is possible due to unfavourable sample preparation in relation to the different wood anatomies. The presence of a large number of uncut cells can lead to wrong results due to the inaccessible pores. Another method involves nitrogen adsorption with BET analysis to characterize pores in the 1–100 nm range. These adsorption methods do not, however, provide any information about pores larger than mesopore size, which have been shown from electron microscopy images to be present in hemp shiv samples. Some studies have been carried out using polar or non-polar liquids as the displacement medium. Nguyen *et al*. [22] studied the inter-porosity of hemp shiv using the pycnometer method, with toluene as the fluid filling. Pure hemp shiv aggregates possessed an inter-porosity of 60% while fibrous hemp shiv had an inter-porosity of 76.6%. Manger [23] concluded that most of the total porosity measurements are variations on bulk volume/grain volume or bulk density/grain density approaches, and the absolute porosity measurements are made by absorption methods employing different fluids or gases. However, the extent to which the cell wall density changes as a function of the sample preparation has not yet been well clarified. Mercury intrusion porosimetry (MIP) is another measurement technique for the determination of pore size distribution. It can be used to characterize pores from a few nanometres to a hundred micrometres in diameter. However, the drawbacks of MIP include the risk of crushing the sample with the high pressures required for analysis. In addition, it cannot distinguish between inter-particle and intra-particle porosity, especially for powdered samples. Finally, the MIP method only determines the diameter of the pore throat, not the actual pore size for ink bottle pores with narrow points of connection, which has the tendency to overstate smaller pore sizes at the expense of larger pore sizes. Thus, the pore size distribution given by MIP can, where ink bottle pores are present, tend to be skewed towards the smaller pores. The total porosity, however, given by this technique, can be considered to be valid as, by the time the highest intrusion pressure has been achieved, either all pores will have been intruded or the pore walls will have been crushed, and their equivalent volume effectively intruded (down to the effective limit of MIP of approximately 3.5 nm).

In this study, hemp shiv was fully characterized by examining cell wall microstructure, porosity and density using different approaches. The microstructure of hemp shiv was studied by SEM and confocal microscopy (CM). The porosity of hemp shiv was fully studied and characterized by employing MIP. The cell wall density was evaluated by helium pycnometry, MIP and the Archimedes’ method.

## 2. Material and methods

The hemp shiv (*Cannabis sativa* L.) used in this study belonged to the herbaceous species originating from Central Asia and was sourced from industrial hemp grown and harvested in north west France by the CAVAC cooperative in 2015. The hemp shiv was produced by a mechanical de-fibring processing of removing the fibre, chopping, grading and de-dusting. During characterization of the shiv, samples from the same batch of hemp shiv from the same field plant were compared. The particles of hemp shiv used in this study were prepared with a mean length of 7.6 mm and a mean width of 2.4 mm.

For measurements performed in a dry state, the hemp shiv was dried in an oven at 60°C until a constant mass was reached (i.e. mass variation lower than 0.1% between two consecutive weighings for three consecutive weighings within a 24-hour period), then it was cooled to ambient temperature in a sealed container. The bulk density of hemp shiv is about 85–90 kg m^–3^. The protocol followed was based on that developed by the RILEM Technical Committee 236-BBM [24], which is a state-of-the-art report that reflects the current knowledge on the assessment of the chemical, physical and mechanical properties of bioaggregate and vegetal concrete, with the following variations: the aggregates were placed in a transparent plastic cylinder 94 mm in diameter and 204 mm in height. The quantity of aggregate was adjusted to be more or less half the volume of the container. The cylinder was upended ten times. The level was marked using a cardboard disc and the volume was measured with water. The transparent cylinder allows the technician to check that no bridges develop within the aggregate which would produce a void with a corresponding reduction in measured density. If this does occur, the cylinder is upended again or the measurement is restarted. The test was repeated three times for each aggregate. The particles of hemp shiv were cut into a mean length of 3.6 mm and a mean width of 1.4 mm to study the effect of size on the density test. The density of hemp fibre obtained from the CAVAC cooperative (France) was compared with the absolute density of hemp shiv using a liquid displacement method.

A scanning electron microscope (JEOL SEM-6480LV, Tokyo, Japan) and a field emission scanning electron microscope (JEOL FESEM6301F) were used for the microstructural analysis of the hemp shiv. All images were taken at an accelerating voltage of 10 kV. The sample surfaces were coated with a thin layer of gold using an HHV500 sputter coater (Crawley, UK) to provide electrical conductivity sending electrons to earth.

The samples of hemp shiv used for binocular microscopy (Zenith XSZ-107BN, UK) were oven-dried at 60°C for three days prior to preparation. The oven-dried material was placed in a tinfoil container and immersed in a two-part resin mixed with a blue dye. The resin was a low viscosity resin with a setting time in excess of 24 h at room temperature. The container was placed in a glass vacuum desiccator and held in a vacuum for 48 h in order to ensure maximum penetration of the resin into the pores. After the resin had set, thin sections of approximately 30 µm were prepared and mounted onto glass slides.

For CM sample preparation, the resin-embedded specimens were trimmed using an ultra-microtome with a glass knife. The slices were observed at room temperature with a confocal microscope (LSM880, Carl Zeiss) using single-track, triple-channel imaging with 405-, 488- and 543-nm laser lines.

For transmission electron microscope (TEM) sample preparation, small piece of hemp shiv were dehydrated in acetone and embedded in low viscosity SPURR resin (TAAB, UK) with vacuum treatment. Ultra-thin sections were cut with an ultra-microtome using a diamond knife. Subsequently, sections were examined with a JEO_JEM_2100 plus TEM at 120 kV.

A CT scanner (Nikon XT H 225) was used to capture the three-dimensional image of hemp shiv specimens. The scanning parameters were fixed at a voltage of 90 kV and current of 108 µA. The area of each pixel is 2.54 µm^2^. The CT scan data were merged using Avizo software (FEI, Thermo Fisher Scientific). The value of white pixels is 0 which represents the pore (air) phase, while the value of blue pixels is 255 which represents the hemp shiv cell wall phase.

Gas pycnometry was carried out using the automatic pycnometers Ultrapyc 1200e (Co. Quantachrome, USA) and AccuPyc 1330 (Micromeritics, UK). Helium was employed as the displacement gas. The cell wall density was calculated from the measured cell wall volume and the known mass of the samples. The apparatus had a 10 cm^3^ cell for AccuPyc and 15 cm^3^ cell for Ultrapyc 1200e. Measurement of the sample volume was achieved by filling the sample cell with helium to the required filling pressure. Then the gas expanded in the expansion cell and the final pressure at equilibrium was recorded (*P*_f_). The volume of the sample was calculated according to the following equation:

2.1 $$V_{\mathrm{sample}}=\frac{V_{\left(\mathrm{samplecell}\right)}-V_{\mathrm{expcell}}}{(P_{r}/P_{f})-1},$$

where *V*_sample_ is the volume of the sample (cm^3^); *V*_sample cell_, the volume of the sample cell (cm^3^); *V*_exp cell_, the volume of the expansion cell (cm^3^); *P*_r_, the run fill pressure; and *P*_f_, the final pressure (psi). The volume of the sample (*V*_sample cell_) and volume of the expansion (*V*_exp cell_) cells were determined by calibration and automatically stored in the set-up parameters.

Different operating conditions have to be defined by the operator before the analysis. In this test, the number of purges and procedures were fixed at ten. Standards for the volume calibration (calibration ball purchased from Micromeritics, *V*_cal_ = 6.371684 cm^3^) were used at 25°C. The experiment was performed by using the cell with a 75% filling ratio. The results showed the density values and standard deviations (corresponding to the ten repetitions for each analysis).

Archimedes’ method was used to measure the absolute density of hemp shiv. Canola oil with a density of 0.92 g cm^−3^ and acetone with a density of 0.79 g cm^−3^ were used for displacement liquids instead of water in order to avoid absorption of water during the experiment and to ensure that the hemp shiv sinks. An electronic balance was used to weigh hemp shiv. Weights were measured to the nearest 0.0001 g. The hemp shiv was cut into small pieces and bundled together with hemp fibre. The dried sample was immersed into liquid and placed into the vacuum chamber. The valve was slowly opened and the air bubbles were pulled from the sample. After a period of time, the bubbles decreased in number and size. The time for the total evacuation of the air from the sample was dependent on several factors including the pore structure of the sample, its size and the power level of the vacuum. Once the bubbling had stopped, the vacuum was released by opening the relief valve. In this experiment, the vacuum was maintained for a period of 8 h. The sample was first weighed in air and its weight recorded as *W*_fa_. The weight of the sample was then recorded in the displacement liquid after vacuum treatment as *W*_fs_. The absolute density (*ρ*_A_) of the sample was calculated using the following equation:

2.2 $$\rho_{A}=\frac{\rho_{s}W_{\mathrm{fa}}}{W_{\mathrm{fa}}-W_{\mathrm{fs}}},$$

where *ρ*_s_ is the density of the solvent (g cm^−3^). All measurements were determined at 22 ± 2°C [25].

An Autopore mercury porosimeter (PASCAL, Thermo Scientific) was used to determine the porosity and pore size distribution of hemp shiv. The measurements were undertaken on four samples (between 0.15 and 0.2 g). Particle size distribution (PSD) *f*(*r*) was determined using the Washburn equation. This relates the radius *r* of pores (assumed to be cylindrical) to the imposed pressure *P* as follows:

$$P=\frac{-2\gamma \;\text{cos}\theta }{r},$$

where *γ* is interfacial energy (surface tension) of mercury and *θ* is contact angle of mercury with the material.

Common values of *γ* and *θ* (which assume interfaces involving a gas or vapour phase) are 485 mJ m^−2^ and 140°. While pores are rarely cylindrical, the Washburn equation is generally accepted as a practical method of analysing what are normally very complex pore systems [26]. The results were plotted in two graphical forms. In the first form, the cumulative pore volume was plotted against a logarithmically spaced abscissa, and in the second form, the differential PSD based on the logarithmic differentiation d*v*/dlog*R* was calculated. The mercury in the porosimeter is intruded into the specimen at a rate of 7–28 MPa min^−1^. The test pressure ranged from 0.0001 to 400 MPa. As a result, the ranges of the pore diameter on the cumulative curve and differential curve were 100 and 0.003 µm, respectively. This wide range allows the detection of diverse pore classes along the PSD curve.

## 3. Results and discussions

### 3.1. Microstructure of hemp shiv

**Figure 2.**
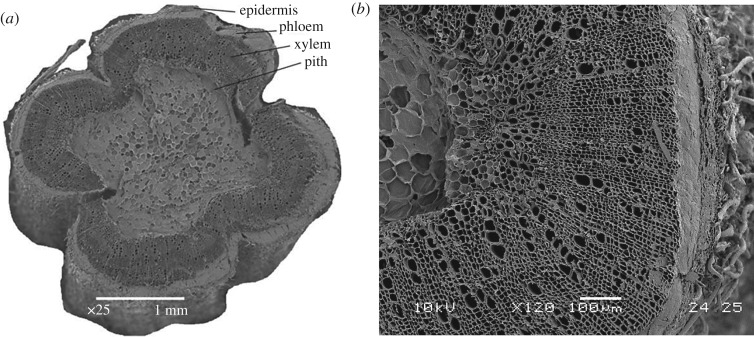
Cross section through hemp stem: (*a*) magnification ×25 and (*b*) magnification ×75.

The cross section of the hemp stem has been identified as the epidermis layer, the phloem layer, the xylem layer and the pith layer from the outside to the centre of its cross section, as shown in figure 2*a*. From the interior to the exterior of the hemp stem, the three zones of pith, xylem and cortex are clearly visible. It can be seen that the stem cross section has an indented shape, similar to a four-leaf clover, which enhances the rigidity of the stem. At higher magnification (figure 2*b*), the radial arrangement of cells in the vascular cambium is visible. In the vascular cambium, there is a subtle boundary between the cells in the outer secondary phloem and the inner secondary xylem. A closer view of the pith reveals the foam-like closed cell structure with some voids at the interfaces between cells. The external surface of the unretted hemp is covered in mechanical fibres (right of figure 2*b*), which are removed during the retting process and subsequently converted into yarns and woven fabrics. Field retting is a major type of retting where harvested hemp stalks are left on the ground for several weeks and the weather is relied on to facilitate the process. The length of the retting process depends on the availability of moisture and air temperature. The vessels exhibit little variation in size and there is no clear pore arrangement in a diffuse-porous distribution. The vessels are mostly solitary although some small groups of adjacent vessels exhibit shared cell walls between them. The vessels are approximately 50–80 µm in diameter and are surrounded by relatively thick cell walls. Thick-walled fibres with a diameter from 1 to 3 µm are located between the vessels. The pore frequency of hemp shiv is around 20.8 vessels mm^−2^. There are no tyloses or other contents in the vessels.

**Figure 3.**
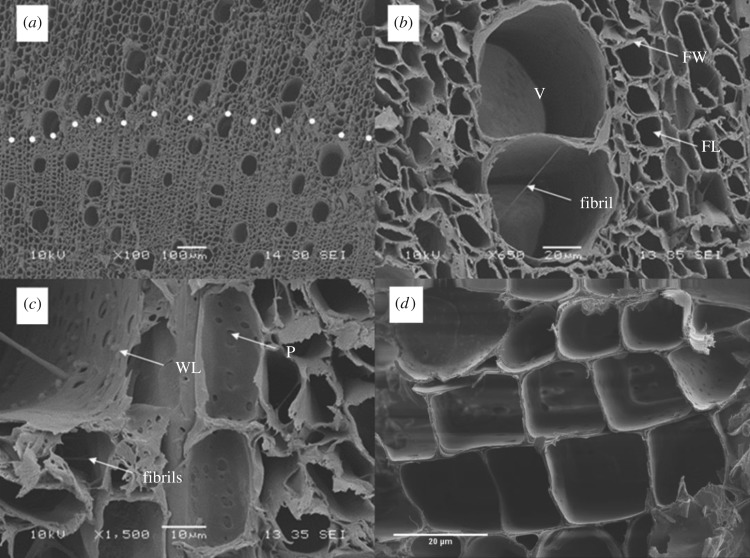
Scanning electron micrograph of the porous hemp shiv, showing (*a*) interface between zones with smaller (lower) and larger (upper) longitudinal cells. (*b*) A close-up view of the vessels shows a bridging fibril in the central vessel. (*c*) The secondary walls of vessels in hemp shiv are overlaid by a warty layer (WL) and several fibrils in the vessels. (*d*) A close-up view of the parenchyma cells seen in the upper section of (*a*). V, Vessel; FW, fibre wall; FL, fibre lumen; P, simple pits; WL, warty layer; dotted line indicates interface.

Figure 3*a* shows a clear interface (white dotted line) between zones with smaller (lower) and larger (upper) longitudinal cells. The large parenchyma cells seen in the upper section of figure 3*a* have an average diameter of approximately 5–10 µm, whereas the size of the parenchyma cells in the lower section of figure 3*a* is much smaller (approx. 1–5 µm). Wall thickness varies from that of fibres to that of axial parenchyma. Several bridging fibrils in vessels can be seen in figure 3*b–d*. A warty layer can just be distinguished on the surface of the secondary wall. The vessel member walls are not always covered in pits. Some walls show prolific vessel to vessel pitting, while others are completely devoid of pits. Bordered pits can be seen within the tracheid cavities. Secondary walls are present and exhibit numerous simple pits. The groups of pits on the walls of the vessel members connect with the ray cells passing behind. The pores are arranged in radial files. The vessel members exhibit profuse vessel to vessel pitting. Small pores below 1 µm in size can be found between cell wall layers.

**Figure 4.**
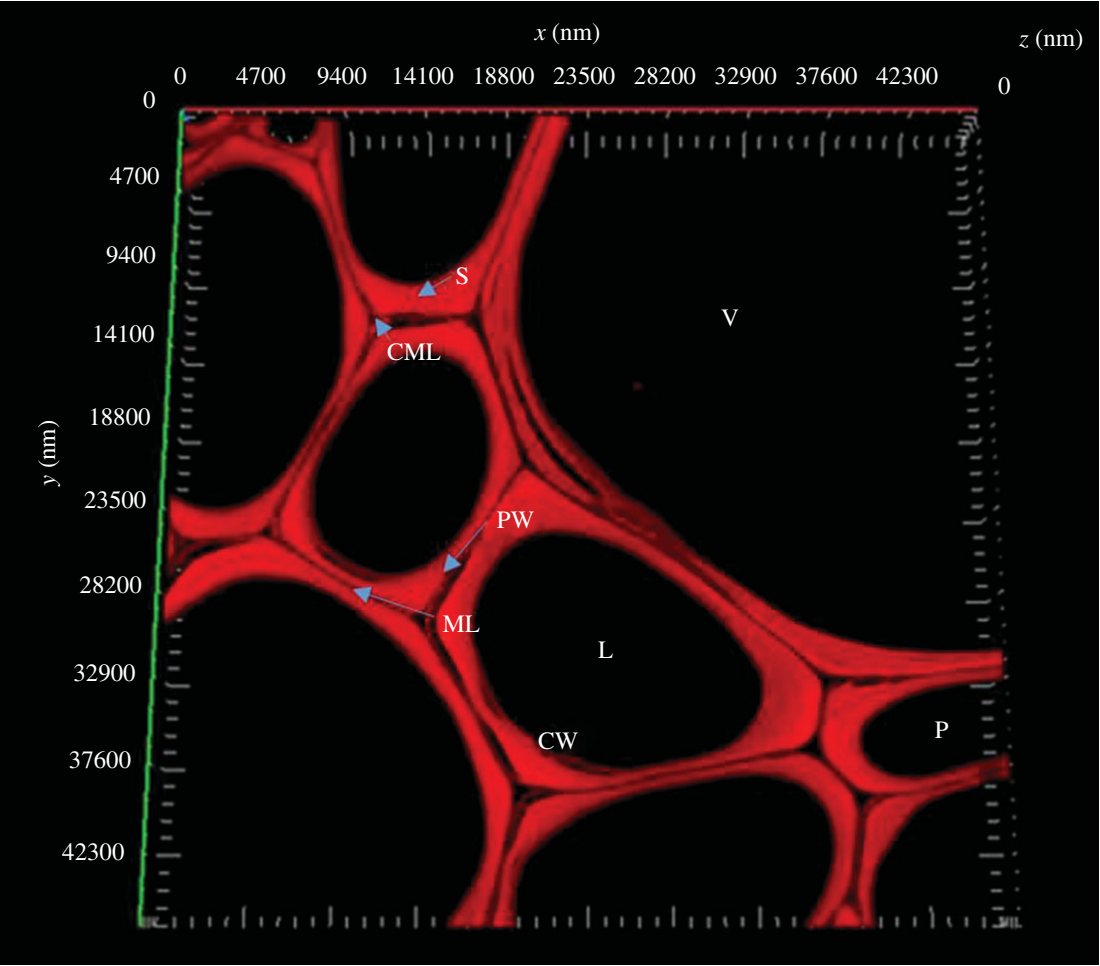
Confocal microscopy showing the cross-section cell wall structure with autofluorescence. V, vessel; S, second wall; P, parenchyma; L, lumen; PW, primary wall; ML, middle lamella; CML, compound middle lamella; CW, cell wall.

Figure 4 shows a cross section of the hemp shiv cell wall, imaged by CM, which is composed of intercellular material, the primary wall and the secondary wall. The secondary wall of the cell and middle lamella are clearly visible in ultra-thin transverse sections of hemp shiv. The dark staining of the middle lamella indicates that it is strongly lignified. Previous reports suggest that the secondary wall of hardwood consists of an outer layer (S1), a middle layer (S2) and an inner layer (S3) [12,27]. These could not be distinguished by CM because of the similarity in their chemical composition and the low resolution of CM. The thickness of the primary plus the secondary cell walls of hemp shiv was found to be in the range of 0.5–2 µm.

**Figure 5.**
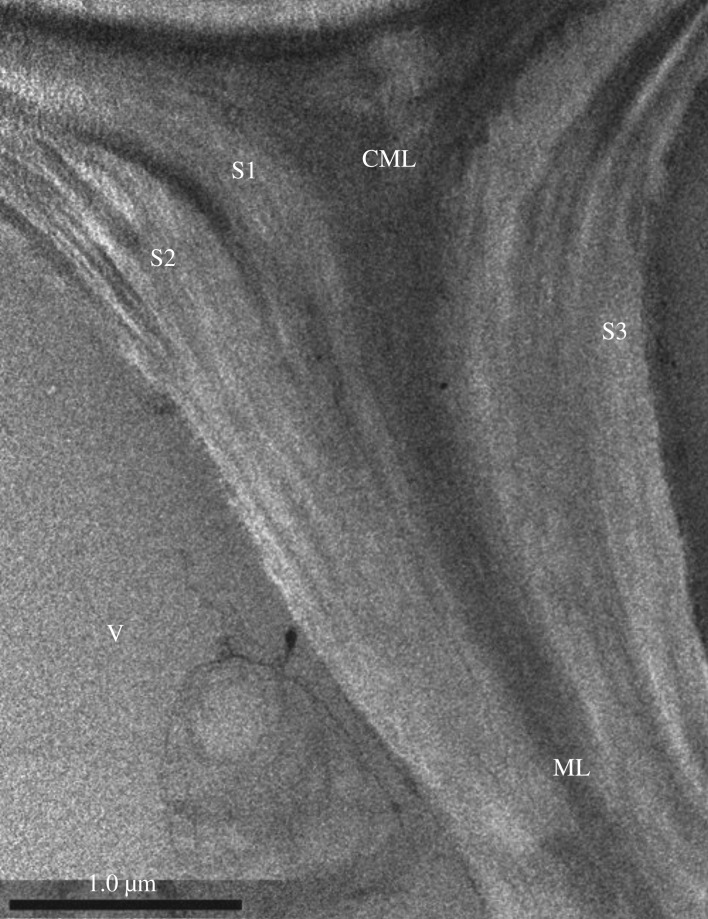
TEM micrographs of cross-section cell wall structure. V, vessel; S1, outer secondary wall of vessel; S2, middle secondary wall of vessel; S3, inner secondary wall of vessel; ML, middle lamella; CML, compound middle lamella.

TEM was used to study the microstructure of cell wall in order to distinguish the layers of the secondary wall, as shown in figure 5. The boundary between primary wall and middle lamella was not clearly distinguishable due to its high density and extreme thinness. Therefore, both the middle lamella and the primary wall were referred to as compound middle lamella. The second wall was divided into an outer layer (S1), a middle layer (S2) and an inner layer (S3). The width of the S1 layer was about 0.2 µm. The S2 layer accounted for the largest proportion of the cell wall. The average thickness of the S2 layer was 0.6 µm. The cell wall also contained an S3 layer that was very thin and not clearly visible.

**Figure 6.**
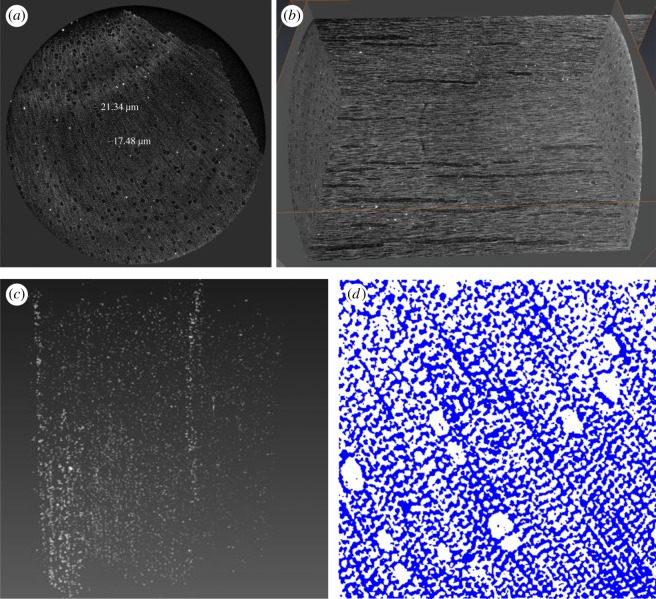
CT scanning measurement data: (*a*) volume rendering of a hemp shiv specimen cross section, (*b*) internal structure, (*c*) distribution of warts and (*d*) cross-section image segmented with the global LA-Kriging method (threshold 125). White is air pore and blue is solid.

**Figure 7.**
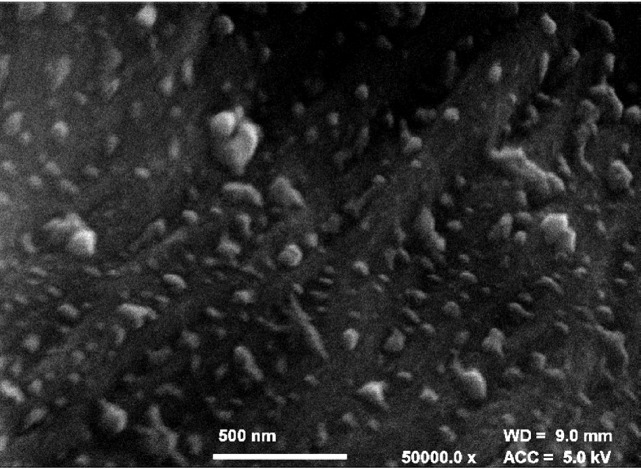
Field emission SEM observations of warts on the surface of cell wall of hemp shiv (radial, longitudinal section) ×50 000.

Figure 6 shows reconstructed sections of hemp shiv from CT scanning. The microtomographic constructions clearly show a high level of anatomical details, revealing the structure of larger vessels, ring boundaries, ray cells and tracheids. Figure 6*a* is a cross section of hemp shiv, which clearly shows there are some macropores with sizes between 20 and 50 µm in the hemp shiv. Figure 6*d* shows a cross-section image of hemp shiv segmented with the global LA-Kriging method [28,29] (threshold 125), which clearly shows the connection between the pores. The pore size and pore size distribution of CT data are in good agreement with SEM images (figure 3). Figure 6*b* shows the microstructure of the inside of hemp shiv. It indicates that a large number of vessels are distributed throughout the hemp structure. The vessels are approximately 100 µm in diameter. Vessels tend to be distributed throughout the hemp shiv rather than preferentially occurring close to growth ring boundaries. Smaller hemp features, such as tracheids and fibres, are an order of magnitude smaller than vessels and require high resolution X-ray microtomography to see them. The CT images give a skeletonized view of the hemp shiv structure showing the orientation and connectivity of the vessels and tracheids. In addition, the full three-dimensional dataset shows the distribution of warts in the hemp shiv after extracting the lower density cell wall. Warts are generally developed in the inner most layer of the wood cell wall, called the warty layer. They are composed of high concentrations of lignin [30]. Figure 6*c* reveals the presence of a warty layer distinct from the S3 or S2 layer. The larger warts and aggregates with a spherical shape become clearly visible. They are not evenly distributed within the hemp shiv. The warts almost cover all the vessel and tracheid cell walls. They have a higher density compared to the cell wall. Evidence has indicated that lignin has a more condensed form in the warty layer than in other parts of the cell wall [30]. They have a wide range of particle size distribution from a few hundred nanometres to a few micrometres. They were also observed in the SEM image (figure 3*c*) and field emission SEM image (figure 7). This is the first report of a warty layer existing in a hemp shiv specimen.

Figure 7 shows the presence of a distinct warty layer with wart diameters in the range 50–200 nm. Large warts consist of an agglomeration of small ones. The warty layer is a remnant of the autolysed protoplast of the tracheal element and also the result of caving in of the S3 layer of the secondary wall inside the tracheid [30].

### 3.2. Porosity and pore size distribution

**Figure 8.**
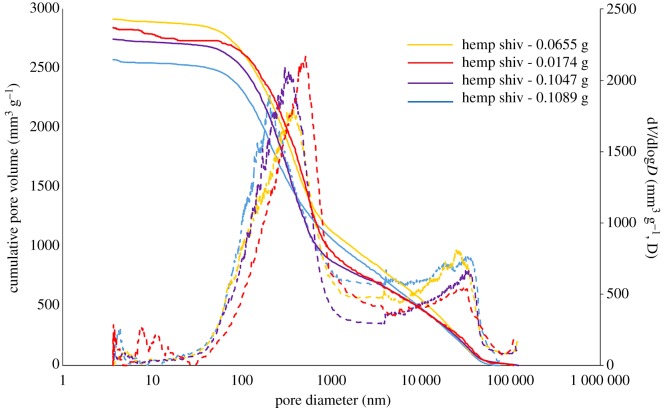
Cumulative pore volume and pore size distribution of hemp shiv with different sample masses determined by mercury intrusion porosimetry.

Table 1 shows the total mercury intrusion volume, total porosity and median pore diameter of hemp shiv studied by MIP measurement. The total intrusion volume of hemp shiv is on average 2.415 ± 0.104 mm^3^ g^−1^ and the total pore surface area is 57.61 ± 7.44 m^2 ^g^−1^. The total accessible porosity of hemp shiv is 76.67 ± 2.03%. A possible compression of the samples, due to the applied high pressure during the MIP measurements, would influence the distribution of measured pore volumes. The hemp shiv used is the woody core of the hemp plant stalk (also known as hurd), as shown in figure 2*b*. Plotze *et al*. [31] showed that some wood samples (white lauan, Afzelia, Macassar ebony, Gaboon, beech, False acacia, Ramin and yew) have a very small sample compression of less than 5% of the measured cumulative pore volume. A further check was carried out by using varying amounts of hemp shiv, ranging from 0.0174 to 0.1089 g, for porosity and pore size distribution tests. No significant difference in porosity and pore size distribution was found among different amounts of sample (table 1 and figure 8). In addition, the pore size distribution measured by MIP shows a good agreement with the results of SEM (mentioned below). It indicated that the applied high pressure during the MIP measurements has little influence on the pore distribution of measured pore volumes. In this study, the common skeletal density of 1.47 g cm^−3^ for the hemp shiv cell wall has been used to calculate the inaccessible porosity [19]. The results showed that the inaccessible porosity is about 0.46 ± 0.18%. It indicates that there is only a small proportion of tiny pores or closed pores, which cannot be accessed by mercury. It is important to keep in mind that mercury porosimetry has the limitation of pore size (diameter between 3 nm and 100 µm). As shown in figure 2*b*, there are no voids in hemp shiv bigger than 100 µm. Thus, this technical restriction is not an issue for the test.

**Table 1.** Some porosity characteristics with ±standard errors for hemp shiv data as determined by mercury porosimetry within the pressure range of 0.0001–400 MPa.

hemp shiv (g)	total intrusion volume (cm^3 ^g^−1^)	total pore area (m^2 ^g^−1^)	median pore diameter (µm)	porosity (accessible) (%)	porosity (inaccessible) (%)
0.1047	2.429	49.81	0.52	77.93	0.24
0.0174	2.369	66.45	0.57	73.45	0.46
0.0655	2.574	63.49	0.54	78.78	0.40
0.1089	2.289	50.69	0.42	76.51	0.75
	2.415 ± 0.104	57.61 ± 7.44	0.51 ± 0.056	76.67 ± 2.03	0.46 ± 0.18

Figure 8 shows cumulative pore volume and the pore size distribution curves for hemp shiv with various sample masses. Because of technical restrictions, the measurement of tracheids larger than 100 µm is excluded. Those pores are, on the one hand, important openings for intrusion but, on the other hand, easily accessible with very low or no applied pressure. As shown in figure 2*b*, there are no voids in hemp shiv bigger than 100 µm. Thus, this technical restriction is not an issue for the test. The pore sizes of hemp shiv show a bimodal pore size distribution with two clearly separated peaks. The main pore radius ranges from 0.03 µm to 1 µm with an average intrusion cumulative pore volume of 2.3 cm^3^ g^−1^ and the second pore radius peak is between 20 µm and 80 µm with an average intrusion cumulative pore volume of 0.8 cm^3 ^g^−1^. The intrusion cumulative pore volume in the range of 1 µm–20 µm pores is about 0.5 cm^3^ g^−1^. Nanoporosity is observed for smaller pores of around 3 nm with a lower peak intensity, which is at the limit of the sensitivity of the MIP technique. This could correspond to the micro-voids or cell wall capillaries. Examination of the SEM images (figure 3) shows that the 20–80 µm macro-voids are produced by the vessels. The 0.03–1 µm micro-voids correspond with the pit membrane voids, pit apertures and other small voids between cell walls. The voids in the range 1–20 µm correspond with the parenchyma cells. The MIP results show good agreement with the SEM images. All the samples show a very similar pore size distribution, although there are variations in peak heights between 3 nm and 10 nm.

### 3.3. Absolute density of hemp shiv substance obtained by displacement with helium gas, organic liquid and mercury

Table 2 shows the effect of different displacement liquids on the absolute density of hemp shiv with different particle sizes. The size of hemp shiv showed no effect on the densities obtained. It indicated that the equilibrium values are independent of particle size. With canola oil, a longer vacuum treatment was required to remove all of the air from the cell wall capillaries, but the results showed that the equilibrium value seems to be independent of the viscosity. There were only slight differences in the absolute density of hemp shiv obtained by using different displacement liquids. The absolute density of hemp fibre obtained by displacement with canola oil (around 1.545 g cm^−3^) is much higher than that of hemp shiv (0.977 g cm^−3^) measured by the same technique. It indicates that the microstructure of the cell wall has a significant effect on the density obtained. It also indicates that the displacement liquid achieves a lower penetration in hemp shiv due to the complexity of the pore structure. Repeat experiments in all cases resulted in a maximum observed deviation of 0.5% from the mean density value.

**Table 2.** Absolute densities of hemp shiv and fibre obtained by displacement with different liquids at 23°C.

sample type	displacement liquid	density of liquid (g cm^−3^)	size of shiv/fibre (L × W × H: mm)	number of tests	absolute density (g cm^−3^)
shiv	canola oil	0.92	7.6 × 2.4 × 1	3	0.965
shiv	canola oil	0.92	3.6 × 1.4 × 1	3	0.977
shiv	acetone	0.79	7.6 × 2.4 × 1	3	1.023
shiv	acetone	0.79	3.6 × 1.4 × 1	3	1.034
fibre	canola oil	0.92	7.6 × 0.1	3	1.545

**Figure 9.**
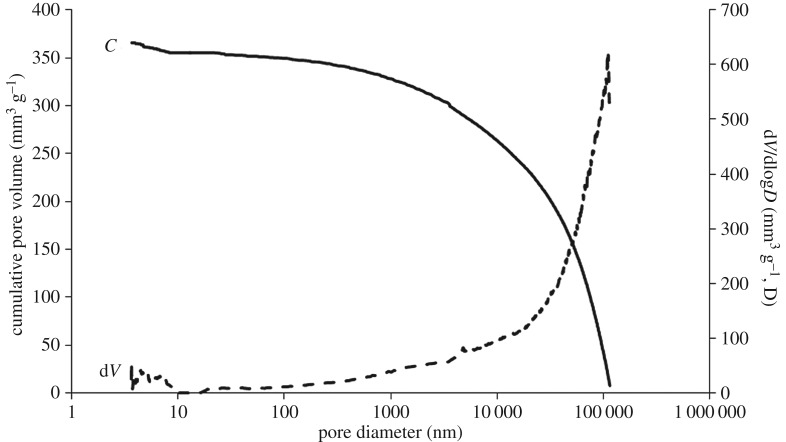
Cumulative pore volume versus pore size distribution for hemp fibre determined by mercury intrusion porosimetry.

**Figure 10.**
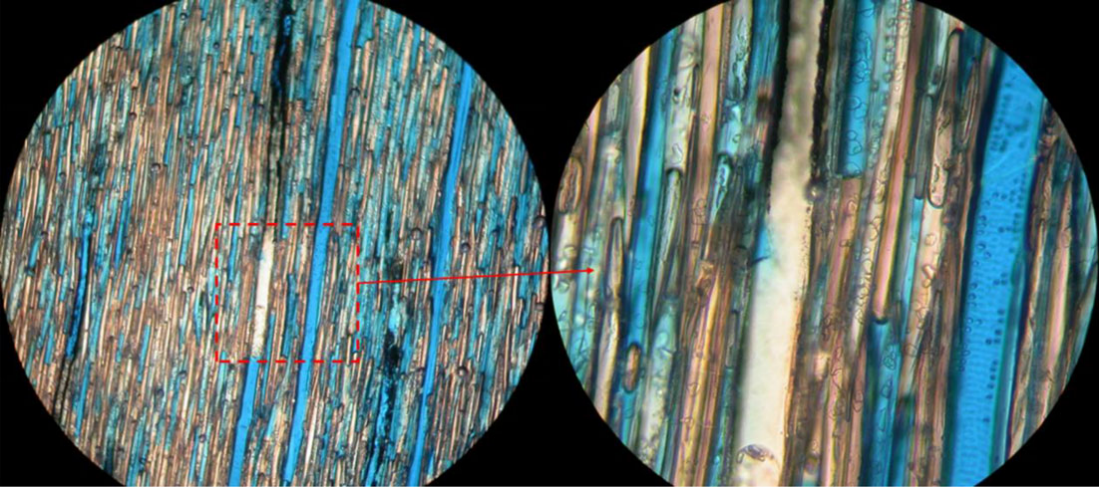
Optical microscopy for colour resin penetration effect in the vessels.

The average absolute densities of hemp fibre obtained from the displacement of liquid appear realistic. The MIP results showed that the hemp fibre contains predominantly large pores (greater than 10 µm), as evidenced by the MIP data in figure 9. The pore sizes of hemp shiv showed a totally different bimodal pore size distribution with two clearly separated peaks, as seen in figure 8. The main pore radius ranges from 0.01 to 1 µm and the second pore radius peak is between 2 and 60 µm. It can, therefore, be assumed that tracheids and cell walls of hemp fibre are freely accessible to canola oils. However, the small pores in hemp shiv (less than 2 µm) are inaccessible to the replacement liquid. The assumption can be confirmed by the colour resin filtration test. Figure 10 clearly shows that the resin cannot reach all the pores in the hemp shiv after the colour resin filtration treatment. Some cells of the lumen and nearly all large vessel pores have been filled with resin. However, there is still a high ratio of tracheid cells inaccessible to the resin. Some of them are partially filled with resin. As a result, the volume of hemp shiv determined is overstated, and consequently the calculated density is understated.

Table 3 presents the absolute densities of oven dry hemp shiv as a function of the sample dimensions. Measurements using helium gas for displacement were undertaken on the assumption that immersion in helium gas gave the true volume of the hemp shiv. The results clearly show that the true densities of hemp shiv obtained from gas replacement are similar to the absolute density results obtained from liquid replacement. This is primarily due to the inaccessibility of some uncut cell lumens or cell walls due to the complexity of the cell wall microstructure. The results also showed that the sample size has no significant effect on the calculated density value.

**Table 3.** Absolute density of hemp shiv, obtained by displacement with helium gas at 23°C.

displacement gas	size of shiv (L × W: mm)	number of determinations	absolute density of hemp shiv (g cm^−3^)	density s.d. (g cm^−3^)
helium	7.6 × 2.4	10	0.947	0.0023
helium	1.6 × 0.4	10	0.958	0.0031

It can, therefore, be considered that the displacement of gas or liquid methods is not suitable to measure the density of hemp shiv without special sample preparation, such as high temperature drying or chemical surface treatment. They will give a falsely low density due to the presence of inaccessible pores.

We already know that pressure can push liquid into small pores without special sample preparation. Thus, the real density of the hemp shiv can be measured only by MIP, if there is no sample compression or collapse under pressurization. Table 4 presents the bulk densities and absolute density of hemp shiv and fibre measured by MIP. The absolute densities of hemp shiv obtained by MIP are significantly higher than the densities obtained by the displacement of canola oil or acetone compared with the displacement of liquid measurement. However, the density of hemp fibre obtained by MIP (1.519 g cm^−3^) is slightly lower than the density obtained by the displacement of canola oil (1.545 g cm^−3^). The results indicate that the mercury has intruded into the small pores in the hemp shiv at high pressure. There are no extra pores that can been filled with mercury at high pressure. The pores in hemp fibre can be fully filled by replacement liquid without extra pressure due to their large pore size. The absolute density of hemp fibre being higher than that of hemp shiv can be explained by the different ratio of three main components in hemp shiv and hemp fibre. Although the hemp shiv and hemp fibre mainly consist of lignin, hemi-cellulose and cellulose, the ratio of the chemical compositions between hemp fibre and hemp shiv is slightly different. The hemp fibre contains less lignin (2–5%) than hemp shiv (19–28%) [32]. The specific density of these three chemical components is slightly different. Lignin has the lowest specific density (1.26–1.41 g cm^−3^) compared to cellulose (1.53–1.57 g cm^−3^) and hemicelluloses (1.50–1.54 g cm^−3^) [32–34]. Thus, the absolute density of hemp fibre is slightly higher than that of hemp shiv. Stamm [35] measured the cell wall density of 10 wood species using three types of displacement media. The measured density ranged from 1.46 to 1.55 g cm^−3^. Kellogg & Wangaard [36] reported that the cell wall density for wood species ranged from 1.46 to 1.53 g cm^−3^. According to the chemical composition of hemp shiv, the cell wall density of hemp shiv should be similar to the literature values for wood species. It means that the MIP method can give a realistic absolute density of hemp shiv without a special sample pretreatment. Furthermore, according to the bulk density (0.32 g cm^−3^) of hemp shiv from MIP data, the porosity of hemp shiv can be determined to be approximately 67% by using the absolute density of displacement of canola oil (0.97 g cm^−3^). Compared to the porosity of hemp shiv obtained from MIP (78%), it can be deduced that around 10% of the pores in hemp shiv are inaccessible to the displacement of canola oils.

**Table 4.** Absolute density of hemp shiv substance obtained by displacement with mercury at 23°C.

sample type	displacement liquid	size of shiv/fibre (L × W: mm)	bulk density of hemp shiv/fibre × (g cm^−3^)	absolute density (g cm^−3^)
shiv	mercury	7.6 × 2.4	0.306	1.443
shiv	mercury	1.6 × 0.4	0.321	1.454
fibre	mercury	7.6 × 0.1	0.984	1.519

## 4. Conclusion

The scanning electron microscope observations of hemp shiv showed distinctive microstructures. The vessels exhibit little variation in size with no clear pore arrangement, resulting in a diffuse-porous distribution. The vessels are mostly solitary although some small groups of adjacent vessels exhibit shared cell walls between them. The vessels are approximately 50–80 µm in diameter and are surrounded by a relatively thick cell wall. Thick-walled fibres are located between the vessels with a diameter from 1 to 3 µm. The confocal images clearly showed the secondary wall and middle lamella in the ultra-thin transverse sections of hemp shiv. The dark staining of middle lamella indicated that it was strongly lignified. The TEM showed the second wall was divided into an outer layer (S1), a middle layer (S2) and an inner layer (S3). CT tomography revealed more detail of the pore shape and pore connection structures of hemp shiv and showed a three-dimensional structure of warts in the hemp shiv. The presence of a warty layer in hemp shiv is reported for the first time. The average accessible porosity of hemp shiv is 76.67 ± 2.03% by MIP measurement. The combination of several techniques and comparison of the results of cell wall structure provides an insight into the complex pore system of hemp shiv, and potentially other similar bio-aggregates across a wide range of pore size distributions, including flax shiv, rape shiv and corn cob. Methods using the displacement of gas or liquid are not suitable for measuring the density of hemp shiv without special sample preparation, such as high temperature drying or chemical surface treatment. They will give a falsely low density due to the presence of around 10% by volume of inaccessible pores. The MIP method can give a realistic real density and porosity of hemp shiv without special sample preparation and is recommended as the appropriate technique for characterization of the pore structure of hemp shiv and other similar bio-aggregates.

## Data accessibility

The datasets supporting this article can be found in the following link: http://dx.doi.org/10.5061/dryad.8t8v6 [37].
